# Delivering the Message: Translating mRNA Therapy for Liver Inherited Metabolic Diseases

**DOI:** 10.1002/jimd.70078

**Published:** 2025-08-18

**Authors:** Sonam Gurung, Dany Perocheau, Roopkatha Ghosh, Stephen L. Hart, Julien Baruteau

**Affiliations:** ^1^ Great Ormond Street Institute of Child Health, University College London London UK; ^2^ National Institute of Health Research Great Ormond Street Biomedical Research Centre London UK; ^3^ Great Ormond Street Hospital for Children NHS Foundation Trust London UK

**Keywords:** gene therapy, inherited metabolic diseases, lipid nanoparticle, liver, mRNA

## Abstract

mRNA encapsulated in lipid nanoparticles (LNPs) provides a dual revolution in the field of gene therapy. mRNA brings fleeting efficacy and the possibility to adjust the therapy to clinical needs. LNP, as a non‐viral vehicle with flexible organ‐targeting, overcomes most immune complications of viral gene therapy. mRNA‐LNP has rapidly progressed from preventive medicine and vaccine applications to therapeutic use, especially in inherited metabolic diseases (IMDs). Given their natural tropism for liver uptake, this platform has been utilised successfully in numerous preclinical programmes. Early phase clinical trials are recruiting to assess safety and efficacy in liver IMDs. Here, we provide the latest update on mRNA and LNP technologies, preclinical studies and clinical trials targeting IMDs, safety considerations with a spotlight on infusion‐related reactions and safety modelling. We discuss the future directions of therapeutic mRNA‐LNP in IMDs and the right clinical use of this adjustable therapy, still to be defined. The versatility of this technology is appealing, with multiple clinical applications as bridge, long‐term cure, rescue, or adjuvant therapy. mRNA‐LNP for gene editing/insertion is an alternative approach for one‐off cure. Translating various successful preclinical programmes in patients remains an unsolved limitation. mRNA‐LNP can be tuned according to the patient's needs and is the next step in personalised medicine and individualised gene therapy.

## 1 Introduction

RNA‐based therapies exploit RNA interference (RNAi), antisense oligonucleotides (ASO) or messenger RNA (mRNA) to modify gene expression and alleviate disease phenotypes. They represent more than 400 programmes, 35 approved drug products and $60 billion in market capitalisation, a figure expected to double in the next 5 years [1, 2, 3]. Among these different RNA technologies, mRNA is the one showing the most rapidly expanding growth, targeting mainly non‐cancerous rare diseases [3, 4, 5, 6, 7]. One third of these mRNA programmes has now reached clinical stage [1]. Uplifted by the unprecedented success of mRNA vaccines against severe acute respiratory syndrome coronavirus 2 (SARS‐CoV‐2), mRNA‐based therapeutics have demonstrated their groundbreaking ability to act as an efficacious and safe technology platform for human use [8, 9]. Building on decades of research since mRNA discovery in 1961, enhanced stability, safety and ribosomal translational efficacy have permitted the use of mRNA as a therapeutic modality in humans [4, 5].

Inherited metabolic diseases (IMDs) are a group of rare monogenic disorders with a cumulative reported incidence of approximately 1 in 2000 live births, with variable incidence according to geographic locations [10, 11]. In most IMDs, therapeutic strategies primarily focus on symptom management rather than addressing the underlying cause, resulting in high mortality and morbidity rates and a compromised quality of life [12]. For some liver IMDs, liver transplantation can be a cure at the expense of procedure‐related morbidity and lifelong immunosuppression. Liver‐directed adeno‐associated viral (AAV) gene therapy has been approved for liver monogenic diseases [13, 14, 15] and has progressed to phase III clinical trials for liver IMDs [16]. However, immunogenicity and transient efficacy in the paediatric liver limit its use in patients with the most unmet needs in infantile and juvenile populations [17]. mRNA encapsulated in lipid nanoparticle (LNP) holds promise for liver‐targeting therapies due to its absence of sustained immunogenicity and the natural tropism of some LNP for the liver, facilitated by the opsonisation of LNP by serum proteins, especially apolipoprotein E (ApoE), which mediates hepatocyte endocytosis via very low‐density lipoprotein receptor (VLDLR) [18, 19]. These properties position LNP as a highly effective delivery platform for treating liver IMDs [20]. Building on these advantages, several preclinical in vivo proof‐of‐concept studies have explored mRNA‐LNP therapies for liver IMDs, progressing towards early phase clinical trials [21].

In this review, we discuss the latest developments of mRNA‐LNP therapy and its applications in targeting IMDs at both preclinical and clinical stages. We highlight safety considerations based on recent studies and provide insights into mRNA‐LNP in the future therapeutic landscape of IMDs, assessing their potential as a long‐term solution or bridging therapy.

## 2 The Toolbox: mRNA and LNP Technologies for Therapeutic Use

The clinical development of mRNA therapies has been greatly facilitated by advancements in mRNA engineering [22] and in vitro transcription (IVT), enabling the production of synthetic and chemically modified mRNA that closely mimic endogenous mRNA [23, 24]. IVT mRNA eliminates the need for costly protein manufacturing and allows simple and cost‐effective production [24]. It also overcomes key challenges of gene therapies, offering higher transfection efficiency, bypassing the need for nuclear transport and eliminating the risk of infection or insertional mutagenesis [5].

**FIGURE 1:**
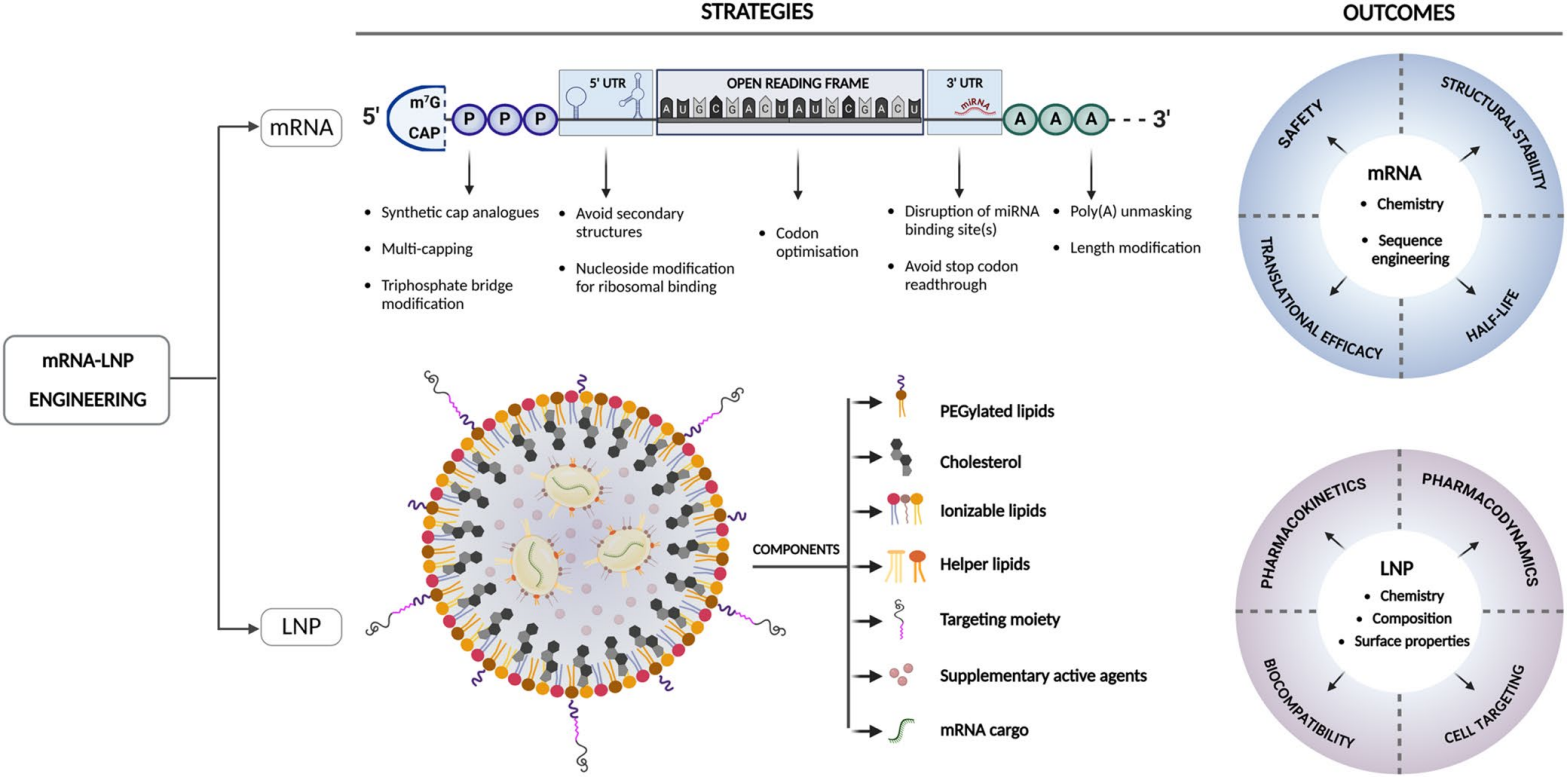
mRNA‐LNP engineering. Each section of the mRNA sequence can be engineered to optimise stability, efficacy and safety. The composition of LNP through lipid chemistry, ratios, and exterior motifs can be optimised to increase targeting, half‐life, and safety. A, adenine nucleotide; LNP, lipid nanoparticle; m^7^G, m^7^methylguanosine; miRNA, microRNA; P, phosphate; PEG, polyethylene glycol; UTR, untranslated region.

Engineered or IVT mRNA is a single‐stranded nucleic acid, which comprises: (i) a 5′ end cap, critical for mRNA stability and translation, (ii) a 5′ untranslated region (UTR), (iii) a coding sequence which starts with the Kozak sequence recognised by the ribosome to initiate translation and ends with a stop codon, (iv) a 3′UTR involved in mRNA stability and translational efficacy and (v) a poly(A) tail to protect the mRNA from degradation (Figure 1) [25]. Chemical engineering with methylation of mRNA nucleotides, such as N^1^‐methylpseudouridine (m^1^ψ) [26, 27, 28] or 5‐methylcytidine [29, 30], improves stability and reduces immunogenicity. This seminal discovery led to Katalin Karikó's and Drew Weissman's Nobel Prize in Physiology or Medicine awarded in 2023 [31]. To limit costly production of modified nucleotides and a theoretical risk of amino acid misincorporation due to modified bases, novel approaches have been developed to synthesise IVT mRNA with unmodified bases and an additional Watson‐Crick base pairing RNA/DNA sequence. The RNA/DNA sequence called Additional Chimeric Element can modulate immunogenicity [32]. Beyond nucleoside chemistry, all IVT mRNA parts can be optimised to increase half‐life, ribosomal entry and protein synthesis. Eukaryotic mRNA requires a 5′‐cap, which protects mRNA from cleavage by nucleases, playing a crucial role in mRNA stability and is essential for the initiation of mRNA translation [33]. This is usually achieved by the incorporation of a modified guanosine nucleotide, 7‐Methylguanosine (^m7^G), linked to the first transcribed nucleotide by a triphosphate bridge [34]. This modification is known as Cap 0 (^m7^GpppN). A methylation of the 2′‐O position of the first cap‐proximal nucleotide forms Cap1 (^m7^GpppN_m_N), whilst a 2′‐O methylation of the next second‐cap proximal nucleotide forms Cap2 (^m7^GpppN_m_N_m_) [33]. However, the production of IVT mRNA can sometimes lead to capping errors where modified nucleosides can sometimes be erroneously incorporated not on the 5′ end but on the 3′ end [35]. These mRNA have shorter half‐lives. To prevent this, an anti‐reverse cap analogue (ARCA) strategy has been developed. Specific modifications of nucleotides will prevent their incorporation at the 3′ end. This is for instance the case by adding a methoxy group (OCH3) at the C3′ position of a ^m7^G (m_2_^7,3′‐O^GP_3_G), which hinders the RNA polymerase from adding the cap in the reverse direction. ARCA are essential for producing high‐quality mRNA and include various cap dinucleotides like m_2_^7,3′‐O^GP_3_G (m(7)3′dGp(3)G or m(2)(7,3′‐)(O)Gp(3)G) [36, 37].

mRNA stability and translation can also be improved by optimising the 5′ and 3′ UTRs. UTR sequences from highly expressed genes such as human β‐Globin [38] are commonly used alongside engineered synthetic sequences designed via bioinformatic tools [39]. Codon optimisation of the mRNA open reading frame coding sequence is crucial for transcriptional activity [40]. The amino acid sequence of the protein of interest can also be optimised to increase the half‐life and the catalytic efficacy of a transgenic enzyme [41, 42]. The poly(A) tail, exposed to exonucleases and deadenylation, can reduce stability and translation capacity if shortened [43]. Poly(A) tail engineering by introducing chemical modifications, site‐specific exonucleases‐resistant modifications, or even multiple branched‐chain synthetic poly(A) tails have significantly increased half‐life [44, 45]. Scalable manufacturing processes and adequate purification methods are essential to obtain translational titres, reproducible batches and non‐immunogenic mRNA‐LNP material.

An effective delivery system is required to encapsulate and protect the mRNA payload, enhance its bioavailability, and ensure its precise delivery [5, 23]. Non‐viral vectors, particularly engineered LNP, are highly versatile delivery systems and the most widely used non‐viral platform for mRNA delivery [46, 47]. LNP were used in the development of the mRNA‐based vaccines against SARS‐CoV‐2 by Pfizer‐BioNTech (Comirnaty) and Moderna (Spikevax), both of which demonstrated over 94% efficacy at preventing symptomatic disease in phase III clinical trials [8, 9]. The advantages of this delivery system include simple formulation, high biocompatibility, high payload capacity, efficient delivery and quick clearance [48]. Advancements in LNP engineering and microfluidic technology have further enhanced their safety, efficacy and scalability and have widened their range of applications [49, 50]. The first drug approvals of LNP delivering siRNA have paved the translational way of RNA‐based therapy [51], currently followed by a rapid expansion of programmes using mRNA as payload, broadening applications for vaccines, protein replacement therapies, or gene editing [7, 52, 53].

LNP have improved significantly from simple liposomes to engineered and sophisticated systems. LNP play the roles of physically protecting their cargo and delivering it into the cytosol via the endosomal pathway [47]. These nanoparticles, sized less than 1 μm in diameter, are formed by four main types of lipids: ionisable lipids, helper lipids, polyethylene glycol (PEG) lipids and cholesterol, and can carry various cargoes including nucleic acids. LNP are taken up by endocytosis via electrostatic fusion with the cell membrane, but also by clathrin‐mediated endocytosis and macropinocytosis [54, 55, 56].

The technology of lipid and polymeric nanoparticles has swiftly progressed in the past two decades. Initial nanoparticle formulations involved the use of cationic liposomes [57]. The development of “pH‐variable lipids” or ionisable lipids came as a breakthrough and an alternative to permanently charged cationic lipids, which were prone to rapid elimination and a toxic profile triggering immunogenicity [47, 51, 58]. Ionisable lipids are positively charged at acidic pH, allowing them to condense mRNA into LNP but are neutral at physiological pH, minimising toxicity. This neutral charge allows a reduction of the protein corona; thereby, facilitating blood clearance and pharmacokinetics, tissue biodistribution and limiting uptake by the reticuloendothelial system (RES) [47, 59]. They become protonated during the acidification process of endosomes and interact with anionic endosomal phospholipids, which facilitates conformational changes, membrane fusion/disruption, endosomal escape and cargo release in the cytosol [60]. Ionisable lipids are recognised as essential for both the encapsulation of negatively charged mRNA and for their optimal release into the cytosol following the fusion of the LNP with the endosomal cell membrane.

Helper lipids are phospholipids acting as stabilisers of the lipid bilayer structure [61, 62]. The two predominant phospholipids used in LNP are 1,2‐distearoyl‐sn‐glycero‐3‐phosphocholine (DSPC) and 1,2‐dioleoyl‐sn‐glycero‐3‐phosphoethanolamine (DOPE). DSPC, a saturated lipid, increases pharmacokinetics; while the unsaturated DOPE, a fusogenic lipid [61], facilitates endosomal escape [63].

Pegylated lipids consist of a hydrophobic lipid anchor, buried into the particle membrane and a hydrophilic PEG polymer on the LNP surface. The PEG domain acts as a shield against surface residual charges, preventing opsonisation in the bloodstream [63], thereby reducing LNP blood clearance [61].

Cholesterol increases pharmacokinetics by reducing the amount of surface‐bound protein and serum opsonins [64]. Modification of the cholesterol tail structure can enhance transfection by facilitating membrane fusion [62, 65, 66]. The biochemical formulation of each lipid component and its respective ratios will influence the LNP size, surface charge, mRNA encapsulation efficacy, tissue targeting and cytoplasmic release. Adding peptide‐targeting ligands at the LNP surface can facilitate on‐target biodistribution [67].

Using mRNA‐LNP to provide long‐term therapy for genetic diseases represents a full change of paradigm compared to the application of this technology for vaccines. If immunisations require the infrequent administration of a limited amount of a highly immunogenic antigenic epitope, treating inherited diseases requires the regular administration of a high dose of a non‐immunogenic transgenic protein. Differences in dose, route of administration and pattern of injection highlight the specificities between these different indications (Table 1). Designing mRNA‐LNP constructs for human translation requires optimisation of various parameters: LNP, mRNA, transgenic protein and manufacturing (Figure 1). Combinatorial high‐throughput screening of these variables can now be performed at large scale [68].

**TABLE 1:** Comparison of mRNA‐LNP technology used for preventive and therapeutic medicine.

		Vaccines	Therapeutics	
Application		Immune protection	Enzyme replacement	Editing
Protein expression		Low	High	
Immunogenicity		Required	None	
Tropism		Not required	Cell‐/‐tissue specific	
Delivery	Route	Intramuscular	Direct or Systemic	
	Pattern	Few times	Long‐term	One‐off
	Dose (per injection)	50–100 μg	300‐900 μg/kg	100‐300 μg/kg
Optimisation		Amplification	Increased signal, stability, half‐life	On‐target biodistribution

**FIGURE 2:**
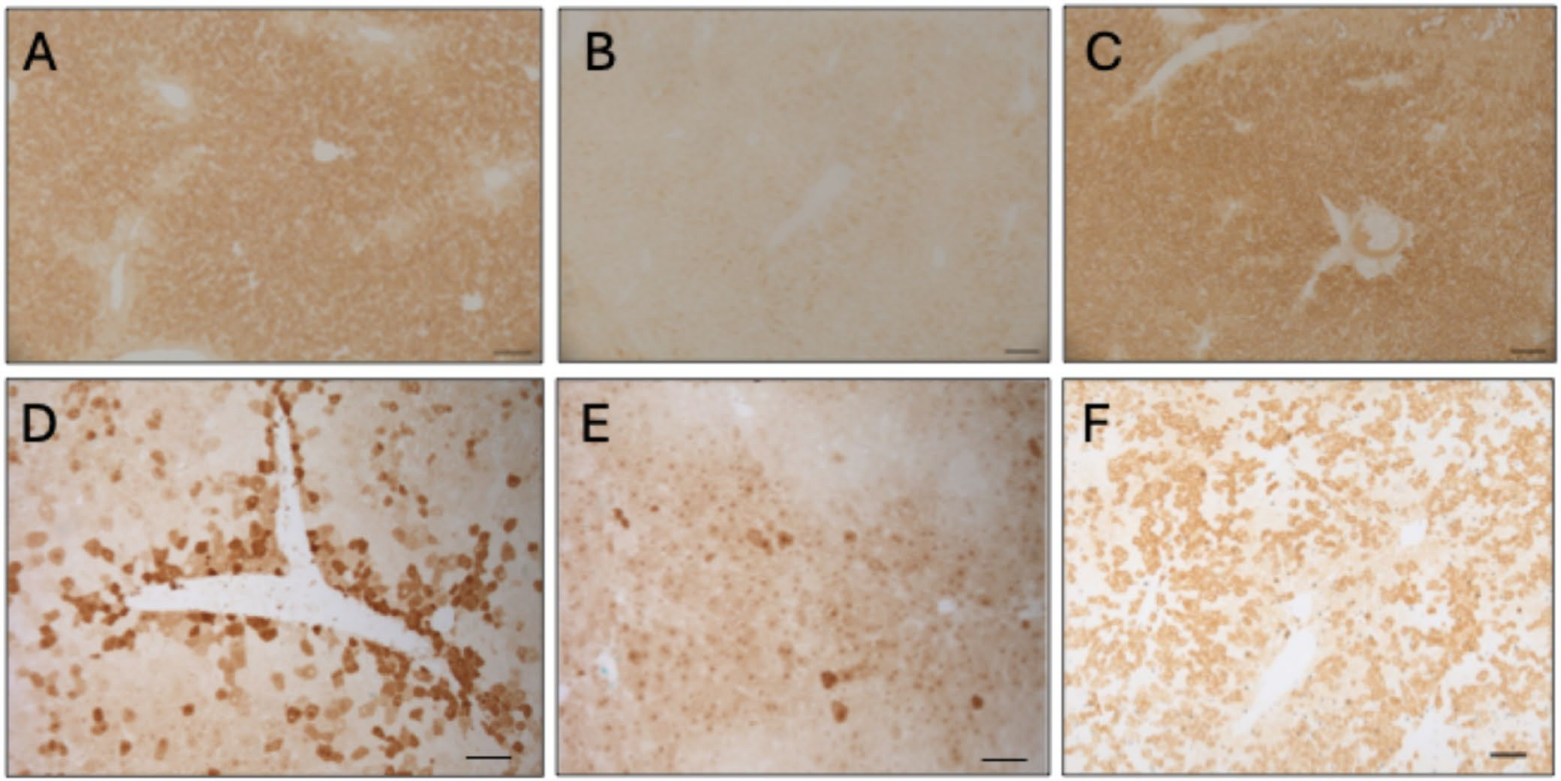
In vivo mouse liver biodistribution of mRNA‐LNP compared to viral gene therapy. Mouse liver immunostaining against the urea cycle cytosolic enzyme argininosuccinate lyase (ASL) for (A) wild‐type and (B–F) hypomorphic ASL‐deficient *Asl* ^*Neo/Neo*^ mouse either (B) untreated, (C) intravenously (IV) *hASL* mRNA‐LNP injected weekly during 7 weeks and sacrificed 48 h after the last injection, (D, E) IV AAV gene therapy injected in (D) adult mouse or newborn pup (E) mouse, or (F) IV lentiviral gene therapy injected in newborn pup. mRNA‐LNP biodistribution is homogeneous and diffuse across the hepatic lobule and liver parenchyma, in contrast with viral vectors, which show heterogeneous transduction. Scale bar: A–C 100 μm, D–F 250 μm.

For human liver applications for IMDs, the differential expression of metabolic pathways through the hepatic lobule called metabolic zonation is of particular importance for cell‐autonomous diseases, in which protein is not secreted [69]. Tackling this challenge requires the transduction of as many hepatocytes as possible, preferably in the metabolic zone where the deficient protein is physiologically expressed. As an example, the urea cycle is expressed in periportal hepatocytes. Interestingly, LNP transfection has been observed either preferentially in periportal hepatocytes in arginase‐ or ornithine transcarbamylase (OTC)‐deficient mice [70, 71] or homogeneously through the hepatic lobule in argininosuccinate lyase (ASL) deficient mice (Figure 2) [72], likely influenced by the LNP formulation and potentially by the pathophysiology of the underlying disease.

## 3 Pre‐Clinical Development of mRNA‐LNP Therapy in Liver IMDs

**FIGURE 3:**
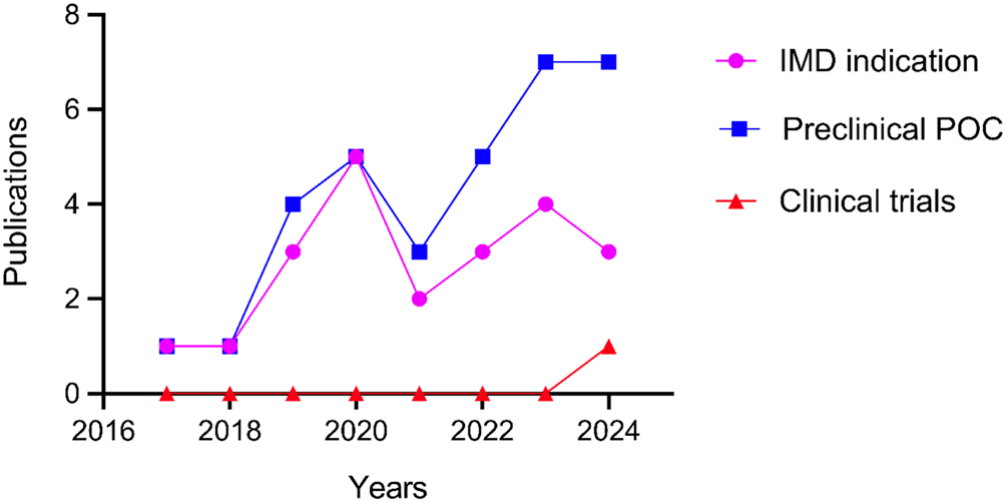
Disclosed targeted inherited metabolic diseases for mRNA‐LNP therapy. The graph represents publications or disclosed reports of mRNA‐LNP therapy for inherited metabolic diseases over time. The first preclinical proof of concept was published in 2016, followed by gradual increase of indications and success, transiently halted during the COVID‐19 pandemic. The first interim results of a phase I/II clinical trial were published in 2024. IMD, inherited metabolic diseases; POC, proof of concept. Data obtained from a Pubmed search using the MeSH terms “mRNA” AND “inherited metabolic diseases” on 15th January 2025.

mRNA replacement strategy using mRNA‐LNP technology has shown great promise in targeting liver monogenic disorders, encompassing a large range of pathways from organic acidemias, fatty acid oxidation disorders, carbohydrate metabolism disorders to urea cycle and amino acid disorders. Over 25 preclinical proof‐of‐concept studies targeting the liver IMDs have been published as of 2025 (Table 2). First publications assessing mRNA therapy in liver IMDs started in 2016 and have gradually progressed, despite a marked stall during the COVID‐19 pandemic (Figure 3). These studies demonstrated sustained efficacy and phenotypic correction in vivo following repeated systemic injections with appropriate dose response, alongside an adequate safety profile. While the preclinical studies are mostly constrained to transgenic mouse models, some have extended the efficacy and safety studies to rats and larger animals such as rabbits and non‐human primates (NHPs), preceding clinical translation [80, 96]. We provide an updated overview of preclinical works for liver IMDs.

**TABLE 2:** Pre‐clinical work implementing mRNA‐LNP.

Disease	Species	Dose	Markers of efficacy	Repeated dosing	Frequency	Toxicity marker	References
Organic acidemias							
Methylmalonic acidemia (MA)	Mouse	0.2 mg/kg	Improved survival, growth, low plasma MMA, low plasma 2‐methylcitrate, low C3/C2 carnitine ratios	—	Weekly	ALP, AST, ALT, IL‐6, Interferon‐ƴ, TNF‐α, IL‐1β	[73, 74, 75]
Propionic acidemia (PA)	Mouse	0.5 and 2 mg/kg, 0.5 or 1 mg/kg	PCC activity, reduction in plasma primary biomarkers (2MC, 3HP, C3/C2 ratio), ammonia reduction	3‐months and 6‐months studies	3‐weekly (3 month study); monthly (6‐month study)	Serum ALT, AST, BUN	[76, 77]
Heme synthesis							
Acute intermittent Porphyria (AIP)	Mouse, rat, rabbit, NHPs	0.2, 0.5 mg/kg	Urinary PBG and ALT excretion, preventing acute induced porphyric attack and recurrent attacks	27 days	3 administrations over 27 days	Serum, albumin, ALP, ALT	[78, 79]
Variegate porphyria (VP)	Rabbit	0.5 mg/kg single dose	Mitochondrial respiratory complex activity, hepatic heme levels, mitochondrial heme A, lipid peroxidation; Systolic blood pressure (hypertension), blood glucose, sciatic nerve amplitude	—	—	ALP, ALT	[80]
Carbohydrate metabolism							
Classic galactosaemia	Mouse	0.5 mg/kg	Gallactose‐1‐Phosphate level (blood and tissue); plasma galactose	8 weeks	Bi‐weekly	—	[78]
Glycogen storage 1a (GSD1a)	Mouse	0.25 mg/kg	Blood glucose levels	52 days	4 doses over 52 days	IFN‐ ƴ, IL‐6, ALT, TNF‐alpha	[81]
Urea cycle and amino acid disorders							
Citrin deficiency (CTLN II)	Mouse	0.5 mg/kg	Hepatic citrulline and blood ammonia post sucrose challenge	—	Single dose		[82]
Ornithine carboxylase deficiency (OTCD)	Mouse	3 mg/kg	Survival, ammonia, orotic acid	70 days	Weekly or bi‐weekly	ALT, AST, cytokine panel	[70, 83]
Argininosuccinic aciduria (ASA)	Mouse	1 mg/kg	Survival, plasma ammonia, ASA, citrulline levels, ureagenesis	3 months	Weekly	ALT	[72, 84]
ASA	Mouse	0.25‐3 mg/kg	Survival benefits	35 days from treatment	Weekly or biweekly		[85]
Arginase deficiency	Mouse	2 mg/kg	Plasma and hepatic arginine and glutamine, plasma ammonia and GAA, ureagenesis	63 days	3 days	Lack of inflammatory infiltrates	[71]
Phenylketonuria	Mouse	3 mg/kg	Plasma phenylalanine: tyrosine ratio, cholesterol, albumin:globulin ratio	336 h	Every 3 days	ALP, ALT	[86]
Tyrosinemia I	Mouse	1 mg/kg	Normalised body weight, serum succinylacetone and Tyrosine levels 21 days post NTBC withdrawal. NTBC supplementation withdrawn 5 days prior to treatment	21 days	Every 5 days	ALT, AST, ALP	[87]
Maple Syrup Urine Disease (MSUD)	Mouse	0.2, 0.5, 1 mg/kg	Survival, reduction in serum leucine	Up to 70 days	Weekly		[88]
Fatty acid oxidation							
Very long chain acyl CoA deficiency (VLCAD)	Mouse	1 mg/kg	Increased tolerance to cold, reduced C16 and C18 acylcarnitines	14 days	5 doses over 14 days	Minimal inflammatory response	[89]
Medium chain acyl CoA deficiency (MCAD)	Mouse	1 mg/kg	Reduced medium chain acylcarnitines, reduced hepatic steatosis, reduced glucose after cold stress	8 days	3 doses		[90]
Oxalate metabolism							
Primary hyperoxaluria type I	Mouse, rat and NHP	0.5–2 mg/kg	Reduction in urinary oxalate	—	Weekly (for toxicology analysis)	IL‐6, CXCL1, ALT, AST, TBIL, CK, C3 and C4	[91]
Bilirubin metabolism							
Crigler Najjar syndrome	Mouse	0.2 mg/kg	Neonatal lethality restored with single dose Repeated dosing restored bilirubin levels in adults (neonatal lethality restored with phototherapy)	Up to 154 days	Every 2 or 3 or 4 weeks	N/A	[92]
Secreted							
α1‐Anti‐trypsin (AAT) deficiency	Mouse	0.5 mg/kg	Reduction in elastase activity, detection of modified human AAT mRNA in liver via in situ hybridisation	N/A	N/A	ALP, AST, CK, GGT and albumin	[93]
Membrane							
Progressive familial intrahepatic cholestasis III (PFICIII)	mouse	1 mg/kg	Restoration of biliary phospholipids secretion, improved hepatomegaly and livery injury, prevents liver fibrosis progression	15 days	twice a week		[94]
Lysosomal							
Fabry disease	Mouse, NHP (safety)	0.2 or 0.5 mg/kg	Restoration of α‐Galactose A (Gal A) activity in heart and liver globotriaosylsphingosine (Lyso‐Gb3) reduction in plasma, heart, kidney, liver and spleen.	3 months	Every two weeks or monthly	Serum albumin, AST, ALP, ALT, GGT, TBIL and TPROT levels in NHPs	[95]

Abbreviations: ALP, alkaline phosphatase; ALT, alanine aminotransferase; AST, aspartate aminotransferase; BUN, blood urea nitrogen; CK, creatine kinase; GGT, gamma‐glutamyl transpeptidase; IL, interleukin; NHP, nonhuman primates; TBIL, total bilirubin; TNF, tumour necrosis factor; TPROT, total protein test.

### 3.1 Organic Acidemias

The first proof‐of‐concept study using mRNA replacement strategy in liver IMDs was demonstrated for methylmalonic acidemia (MMA) [73, 74]. mRNA‐LNP encoding codon‐optimised human methylmalonyl‐CoA mutase (*hMUT*) showed robust hepatic protein expression for at least 7 days after single systemic administration. Long‐term efficacy with repeated administrations was achieved in two MMA murine models, the *Mut* ^*−/−*^ *; Tg* ^*INS‐Mck‐Mut*^ mouse with the *Mut* gene under the transcriptional control of a muscle‐specific creatine kinase (*MCK*) promoter and the *Mut* ^*−/−*^ *; Tg* ^*INS‐CBA‐G715V*^ mouse, a hypomorphic model ubiquitously expressing a mouse orthologous mutation (p.G715V) of an MMA patient mutation (p.G717V). The study demonstrated increased hepatic MUT activity, improved growth, survival and reduced disease‐associated plasma metabolites in both models. Lack of increased toxicity and inflammation markers following repeated dosing demonstrated safety [73, 74]. This programme has been translated into a phase I/II clinical trial (NCT04899310).

Similarly, mRNA replacement therapy was successful in propionic acidemia (PA) [76]. This work highlighted the long‐term therapeutic effect of a combined mRNA therapy. PA is an IMD caused by propionyl‐CoA carboxylase (PCC) deficiency localised in the mitochondrial matrix. PCC is a large heterododecamer enzyme composed of α‐ and β‐subunits (PCCA and PCCB). Although patients have pathogenic mutations in either the PCCA or PCCB subunit, the alternative subunit cannot compensate as two functional subunits are required for a functional PCC enzyme. LNP encapsulating an equimolar ratio of *hPCCA* and *hPCCB* mRNAs was successfully tested in a hypomorphic PA murine model (*Pcca* ^*−/−*^ (A138T) mice). Single IV dosing of 1 mg/kg mRNA resulted in therapeutically relevant protein expression of both PCCA and PCCB subunits, comparable to endogenous protein levels in human liver [76]. Long‐term repeated dosing for up to 6 months demonstrated sustained efficacy and tolerability. This therapeutic candidate was taken forward for a phase I/II clinical trial (NCT04159103; NCT05130437) with interim data published in April 2024 and detailed in the next section [21].

### 3.2 Urea Cycle Disorders (UCDs)

Several independent preclinical studies have demonstrated the efficacy and safety of mRNA‐LNP to replace defective enzymes involved in the urea cycle pathway, such as citrin deficiency [82], ornithine transcarbamylase deficiency (OTCD) [70, 83, 97], argininosuccinic aciduria (ASA) (resulting from argininosuccinate lyase (ASL) deficiency) [72, 85] and arginase deficiency [71, 98]. The LNP encapsulating OTC‐mRNA (LUNAR‐OTC) to target OTCD is currently undergoing Phase II clinical trials [97]. A study in a hypomorphic mouse model of ASA not only showed correction of survival, disease biomarkers and restoration of ureagenesis but also correction of the global liver metabolic dysfunction, including dysregulation of glutathione metabolism [72]. Sustained phenotypic correction was observed following neonatal initiation of therapy. Rescue of juvenile ASL‐deficient mice demonstrated the ability for mRNA‐LNP to reverse a severe pre‐dying phenotype [72]. Indication of neurological advantage following normalised ureagenesis with mRNA‐LNP therapy was also evident, albeit indirectly, in the ASA hypomorphic mouse model with the absence of abnormal neuroimaging findings [84] and in precision‐cut liver slices [99]. mRNA‐LNP corrected ureagenesis and the liver metabolic phenotype in arginase deficiency [71] This enabled subsequent therapeutic benefit on the neuropathology, as observed with myelination improvement preventing leukodystrophy and oligodendrocyte dysfunction in vivo [98]. mRNA‐LNP showed an improved phenotype, particularly in behavioural changes, in a hypomorphic mouse model of citrin deficiency with improved sucrose aversion [82].

### 3.3 Aminoacidopathies

Similarly, multiple independent proof‐of‐concepts of mRNA‐LNP therapy have been shown for phenylketonuria [77, 86], tyrosinemia type I [87, 100] and Maple Syrup Urine Disease (MSUD) with correction of the clinical and biological phenotypes [88].

### 3.4 Porphyrias

Porphyrias are a group of IMDs resulting from a defect in enzymes in the heme biosynthetic pathway [101]. Porphobilinogen deaminase (PBGD) is the third enzyme in this pathway, and its hepatic deficiency results in acute intermittent porphyria (AIP). Patients predominantly display acute neurovisceral attacks due to high production of neurotoxic porphyrin precursors and additional heterogeneous symptoms [102]. The therapeutic benefit of *PBGD* encoding mRNA was demonstrated by the rescue of acute porphyric attacks and prolonged repeated dosing prospectively preventing attacks in AIP mice. The therapeutic efficacy was further demonstrated in a chemically induced porphyrin precursor rabbit model. Importantly, this study showed the translatability of the administration dose from the mouse model (0.5 mg/kg) to rats and larger animals such as rabbits and NHPs, achieving up to 80% of endogenous activity within 24 h post‐dosing with minimal toxicity [103]. The sustained efficacy and tolerability during repeated administration tested successfully in all species demonstrated the therapeutic benefit of mRNA‐LNP. Interestingly, another study in a novel rabbit model of chemically induced Variegate Porphyria (VP) evaluated the therapeutic efficacy of systemic administration of LNP encapsulating *PBGD* mRNA. While VP is resultant from haploinsufficiency of protoporphyrinogen oxidase (PPOX), the seventh enzyme in the heme biosynthesis pathway, *PBGD* mRNA showed efficacy in preventing acute attacks [80].

### 3.5 Energy‐Related Disorders

mRNA replacement strategy has also been successfully implemented in fatty acid oxidation disorders such as very‐long‐chain acyl‐CoA dehydrogenase deficiency (VLCADD) [89] and medium‐chain acyl‐CoA dehydrogenase deficiency (MCADD) [90] carbohydrate metabolism disorders such as classic galactosemia [78] and glycogen storage disease 1a (GSD1a) [81, 104]. Proof‐of‐concept study in a GSD1a mouse model showed efficacy of mRNA therapy in addressing both life‐threatening acute hypoglycemia and chronic risk of hepatocellular adenoma and carcinoma (HCA/HCC) associated with the disease [81]. This GSD1a programme has been translated into a phase I/II clinical trial (NCT05095727).

### 3.6 Lysosomal Disorders

Fabry disease is the most common lysosomal storage disease resulting from lack of functional alpha galactosidase A (α‐GalA) activity causing accumulation of glycosphingolipids. Preclinical study of mRNA replacement strategy demonstrated efficacy in a Fabry disease mouse model and safety in non‐human primates (NHPs). Bi‐weekly administration of up to 4 doses in NHPs showed sustained secreted functional α‐GalA protein and absence of immunogenicity [95]. Preliminary work in Niemann‐Pick disease Type C (NPC‐1) has shown proof of concept with restoration of functional protein expression and reversal of disease pathology in NPC‐1 patient fibroblasts [105].

### 3.7 Oxalate Metabolism

Primary hyperoxaluria type I is a liver IMD where lack of function of alanine glyoxylate aminotransferase (AGT) causes accumulation of glyoxylate, a metabolite converted into oxalate. Oxalate accumulation results in deposition of oxalate crystals causing a severe systemic disease affecting various organs, such as kidney with stones and causing progressive renal failure, bone, heart, skin and retina [106]. Single injection of sequence‐optimised human *AGT* mRNA resulted in AGT protein expression for up to 48 h, and a single dose of 2 mg/kg *hAGT*mRNA‐LNP therapy in a rat model of AGT deficiency, WT mice and Cynomolgus monkeys [91]. Significant reduction in urinary oxalate > 70% was observed in AGT‐deficient mice and rats within 48–72 h with minimal toxic profile at up to 2 mg/kg dose [91].

Other conditions include Alpha 1‐antitrypsin (AAT) deficiency where levels of circulating AAT are significantly lowered due to aberrant protein folding and aggregation in the rough endoplasmic reticulum of hepatocytes [93]. Systemic administration of mRNA therapy in a transgenic mouse model of AAT deficiency led to hepatic uptake and translation into effective protein in mice; with phenotypic improvement [107].

## 4 Clinical Trials of mRNA‐LNP Therapy in Liver IMDs

Following the successes of mRNA‐LNP technology in preclinical models, various phase I/II clinical trials have been announced targeting liver IMDs (Table 3).

**TABLE 3:** mRNA‐LNP clinical trials for liver IMDs.

Condition	Sponsor	Phase	Drug	Status	Clinical trial number
Propionic acidemia (PA)	Moderna	Phase I/II	mRNA‐3927	Recruiting	NCT04159103 NCT05130437
Methylmalonic acidemia (MMA)	Moderna	Phase I/II	mRNA‐3705	Recruiting	NCT04899310
Ornithine transcarbamylase deficiency (OTCD)	Arcturus	Phase I/II	ARCT‐810	Recruiting/completed	NCT06488313/NCT04442347/N CT04416126
Glycogen storage disease 1a (GSD1a)	Moderna	Phase I/II	mRNA‐3745	Recruiting	NCT05095727
Phenylketonuria (PKU)	Moderna	Phase I/II	mRNA‐320	Recruitment complete	NCT06147856
Crigler–Najjar disease	Moderna	N/A	—	No updates	Preclinical

The interim data of the first‐in‐human phase I/II mRNA dose optimisation and extension study trial for PA sponsored by Moderna Inc. (NCT04159103; NCT05130437) were recently published, showing promising results [21]. The study evaluated the safety and efficacy of mRNA‐3927, a dual mRNA‐LNP therapy encoding mRNA for *hPCCA* and *hPCCB* delivered systemically [76]. Sixteen participants were allocated to five different cohorts. Cohort 1 received a dose of 0.3 mg/kg every 3 weeks, while cohorts 2–5 received treatment every 2 weeks at doses of 0.3, 0.45, 0.6 and 0.9 mg/kg respectively. A total of 321 doses had been administered at the data cut‐off. Safety data showed no dose‐limiting toxicity, with the mRNA injections being generally well‐tolerated, with treatment‐related adverse events reported in 15/16 patients and serious adverse events in 8/16 patients. The severe adverse events were not attributable to the drug itself, especially a grade 3 pancreatitis attributed to the underlying genetic disease by the Data Safety Monitoring Committee. Infusion‐related reactions (IRRs) were a common finding observed in 5/16 (31.3%) patients and in 18/346 (5.2%) doses. These IRR manifestations were essentially grade 1 and 2 rashes occurring during the first few doses, then disappearing in most cases in line with a tachyphylaxis effect previously described with liposomal drugs. The IRR manifestation resulted in the withdrawal of one patient from the trial.

A preliminary trend of increased pharmacokinetics with dose escalation was observed from the serum analysis of *hPCCA* mRNA. A 70% reduction in relative risk of metabolic decompensation events (RR 0.3 with 95% CI 0.066–1.315; *p* = 0.0927) showed an encouraging trend, despite limitations from a limited study cohort, lack of a control arm and 2‐year follow‐up completed only for 2 patients from the lowest dose cohort. Another consideration linked to the pathophysiology of this multisystemic disease is that a liver‐targeting mRNA‐LNP can provide a significant but still partial correction of the disease. As illustrated by liver‐transplanted PA patients, a significant production of toxic metabolites from extrahepatic organs still persists, and the risk of acute metabolic decompensations and extrahepatic organ complications, although decreased, remains [108]. Hence, results at study completion will be closely scrutinised. A follow‐up phase III clinical trial will assess the long‐term safety and clinical activity (NCT04159103) and will be key in determining the clinical translational potential of mRNA‐LNP in PA.

Additional clinical trial data have been disclosed in non‐peer reviewed communications. A phase I/II clinical trial on patients diagnosed with isolated MMA using mRNA‐3705 (NCT04899310) and sponsored by Moderna Inc. is recruiting, assessing safety, pharmacokinetics and dynamics with two phases of dose optimisation and dose expansion. *Corporate press releases have disclosed* encouraging initial pharmacodynamics [109], following the dosing of 11 participants and a total of 221 doses administered intravenously. The treatment was reported as well tolerated, with dose‐dependent reduction in metabolite in MMA in certain cohorts, decrease of yearly metabolic decompensation events, and MMA related hospitalisations [109, 110]. *No peer‐reviewed data have been published, and the disclosed information should be interpreted with caution*. A follow‐up extension trial was announced to evaluate long‐term safety, including treatment and follow‐up periods (NCT05295433).

Arcturus therapeutics also reported on phase IA single ascending dose (SAD) study for ARCT‐810, an mRNA therapeutic candidate for OTCD [111]. Thirty healthy adults were enrolled and randomised 2:1 to receive ARCT‐810 at multiple ascending doses or placebo intravenously (NCT04416126). This was followed by a phase 1B SAD study which enrolled 16 adults with metabolically well‐controlled OTCD, randomised 3:1 to receive single doses of the therapeutic candidate or placebo (NCT04442347). An ongoing phase II study has been initiated in OTCD adolescents and adults, with subjects randomised to receive 5 doses or placebo every 14 days (NCT06488313, NCT05526066) [111].

Additional clinical trials announced through press releases include phase I/II clinical trial for GSD1a (NCT05095727) with mRNA‐3745 (actively recruiting) [111], PKU with mRNA‐3210 (NCT06147856) (withdrawn) [109, 111], mRNA‐3139 for OTCD and mRNA‐3351 for Crigler‐Najjar Syndrome (CN‐1) [112].

Overall, various early phase clinical trials are ongoing for liver IMDs, with limited interim results published thus far. First interim results support safety despite a high rate of infusion‐related reactions [21]. A trend for efficacy has been reported but requires study completion to confirm the therapeutic value and its extent.

Interestingly, a bioinformatic model has been published validating this approach to efficiently assist with extrapolation of minimally effective dosing while translating mRNA‐LNP from preclinical animal models into patients [77, 113]. This is of particular interest as each liver IMD is expected to have a different therapeutic threshold of transgenic protein expression.

## 5 Safety and Immunogenicity of mRNA‐LNP

Ensuring long‐term safety for a therapy, which requires lifelong administration, is key. As the technology is gradually being translated to patients, disclosed safety data are currently limited to a few patients who have received mRNA‐LNP for a maximum of 2 years [21]. In the PA trial sponsored by Moderna (NCT04159103; NCT05130437), no dose‐limiting toxicity was observed at the highest dose of 0.9 mg/kg mRNA administered every 2 weeks; although the duration of follow‐up data disclosed remained limited to 6 months maximum in this high‐dose cohort [21].

The immune response described against mRNA‐LNP is mainly based on innate immune response or anaphylactic reactions mediated by either IgE or IgM‐complement. To study mRNA‐LNP related immunogenicity, in vitro (e.g., cell viability assays, incubation with immune cells) and in vivo (e.g., small and large animals) models have been used. Innate immune response can target either mRNA, LNP, or the combination mRNA‐LNP. In clinical trials for IMDs, this innate immune response has not been reported.

Synthetic mRNA can be recognised by multiple endosomal (Toll‐like receptors (TLR) 7,8,9) and cytosolic protein kinase RNA‐activated (PKR), retinoic acid‐inducible gene I protein (RIG‐I), melanoma differentiation‐associated protein 5 (MDA5) and 2′–5′‐oligoadenylate synthase (OAS) innate immunity sensors. These sensors can be triggered by single‐stranded (via TLR7, 8) or double‐stranded (via TLR3) mRNA or LNP. Upon activation, they initiate an inflammatory cascade through myeloid differentiation primary response protein 88 (Myd88)/NFKB (nuclear factor‐κB)—CREB (cAMP response element‐binding protein), interferon regulatory factor (IRF) activating type 1 interferon response [114], chemokines (CXCL1 and CXCL2), cytokines‐mediated response (IL‐6, IL‐10, IL‐12β and IL‐1β), and leucocyte recruitment [115]. Incorporation of naturally occurring modified nucleosides such as (N1‐methyl)pseudouridine, 2‐thiouridine, 5‐methyluridine, 5‐methylcytidine, or N6‐methyladenosine prevents mRNA recognition by TLR3, 7, 8 and precludes the associated innate immune response [113].

Strong evidence has been generated that lipid‐based carriers, especially LNP, participate and trigger the quality and the intensity of the innate immune response, mediated by the NLRP3 inflammasome, IL‐1 and IL‐1 receptor antagonist (IL‐1ra) [116, 117] or IL‐6 [118]. This adjuvant role of LNP in triggering innate immunogenicity has been well documented in mRNA vaccines, particularly with ionizable lipids like SM‐102 from Moderna's vaccine, with species‐specific responses [116, 117].

Hence, both LNP and mRNA, independently or in combination, can trigger innate immune response [116]. Interactions between ionisable lipids and pattern recognition sensors are complex and multifactorial. Further research is required to better unravel these mechanisms and develop safer lipid‐based particles.

In the PA trial (NCT04159103; NCT05130437), infusion‐related reactions were a prominent adverse event reported in 31.3% of patients, essentially during the first administrations. These reactions are analogous to what has been described with other liposomal drugs such as oncolytics, for example, doxorubicin [119], and other RNA‐based therapies, for example, mRNA vaccines [120, 121] or small interfering RNA (siRNA) [122].

A limited number of LNP drugs has been approved so far [51]. In those, the lipid composition and additional conjugated motifs such as PEG retain physicochemical features and biological activity, which enable improved pharmacokinetics and liver‐targeting. However, PEG‐related immune‐induced adverse events are not uncommon and include hypersensitivity, complement activation related pseudo allergy (CARPA) and occasionally anaphylaxis [123]. Those events have been reported with mRNA‐LNP vaccines and LNP‐based RNA therapies.

The immune reactions of hypersensitivity (i.e., rash, dyspnea, chills, chest pain, tachycardia, hypotension, hypertension) and anaphylaxis observed with the COVID‐19 vaccines have been reported with greater incidence compared to traditional vaccines, with an estimated anaphylaxis event of 2.5–5/1,000,000 vaccine doses [120, 124]. This risk was 5000 times higher in known allergic patients [121].

Patisiran (Onpattro) produced by Alnylam, is a double‐stranded siRNA encapsulated in LNP and approved for the treatment of hereditary transthyretin amyloidosis. Hereditary transthyretin amyloidosis is a genetic, progressively fatal disease, causing neuropathy and cardiomyopathy due to the accumulation of amyloid fibrils composed of misfolded transthyretin aggregates. Patisiran, administered intravenously every 3 weeks, acts by inhibiting the synthesis of the transthyretin protein in hepatocytes. Patisiran was approved in 2018 by the FDA, becoming the first type of drug developed with LNP encapsulation of nucleic acids. The most common adverse reactions associated with patisiran are upper respiratory tract infections and infusion‐related reactions, with flushing, abdominal pain, nausea, respiratory symptoms, headache, rash, tachycardia and/or changes in blood pressure. All patients are required to receive premedication based on corticosteroids, acetaminophen and anti‐histamines (H1 and H2 blockers) [125]. In clinical trials, infusion‐related reactions were observed in 19% of patients versus 9% in placebo controls, with 79% occurring during the first 2 infusions and frequency decreasing over time. These reactions led to infusion interruption and permanent treatment discontinuation in 5% and < 1% of patients, respectively [122].

LNP‐mediated hypersensitivity or anaphylaxis adverse events described in populations treated by mRNA‐LNP based vaccines can be triggered by IgE‐mediated classical pathway or IgM‐mediated (IgE independent) reactions. In IgE‐mediated reactions, IgE binds to the mast cells and basophilic granulocytes and will cause the release of histamine, prostaglandins, leukotrienes and tryptase. This can be triggered by anti‐PEG IgE antibodies, for example [126]. Another mechanism is CARPA, where anti‐PEG IgM binds to the LNP and activates the classical complement pathway, with the generation of complement products C3a, C4a, C5a, which act as anaphylatoxins, potentially causing anaphylactic reactions [126]. CARPA reactions can be reproduced in piglets following the injection of PEGylated liposomes or mRNA‐LNP vaccines. This causes anaphylactic shock and pulmonary hypertension, thereby enabling close monitoring of PEG‐triggered hypersensitivity reactions and complement activation in a large animal model [127, 128]. Therefore, current enrolment criteria for clinical trials targeting IMDs can exclude patients with a severe allergic reaction to liposomal or PEG‐containing products, like for the OTC deficiency trial (NCT05526066, terminated as of April 2025) [111].

Autoimmune reactivity, which has been observed in mRNA‐LNP vaccination, has not been described in IMD‐treated patients [115].

## 6 Perspectives on Translational Use of mRNA‐LNP as Therapy for IMD: Bridge or Long‐Term Cure?

With a rapidly growing number of successful preclinical studies and emerging clinical data in liver IMDs, the therapeutic promise of mRNA therapy for these conditions cannot be disputed. Interestingly, its versatility with transient effect and possible modifications of dose or injection frequency provides flexibility in its use, which had not been conceptually envisaged when gene therapy was initially considered and developed as a one‐off cure. This adaptability opens opportunities for liver IMDs.

AAV gene replacement therapy is currently the leading gene therapy technology for liver IMDs with hundreds of clinical trials reported in monogenic diseases [13, 15, 129]. However, major limitations persist such as pre‐existing immunogenicity to AAV capsids resulting in exclusion of patient cohorts, innate and adaptive immune reactions post‐dosing, inability to re‐dose, limited sustained efficacy in the growing paediatric liver, limited transgene packaging capacity and high production costs [13, 129, 130]. Emerging research using integrating vectors such as lentiviral vectors or nuclease‐mediated integration strategies could enable long‐lasting expression due to integration of the therapeutic transgene in the host genome. Additionally, lentiviral vectors have increased payload capacity up to 15 kilobases [131]. But uncertainty remains regarding the long‐term efficacy and safety, with risks of insertional mutagenesis from off‐target integrations [130].

Protein/enzyme replacement therapy allows direct protein delivery bypassing complex cellular transcription and translation. This has been applied in diseases requiring substitution of hormones, enzymes, blood clotting factors and interferons. But protein instability and short half‐life, potential immunogenicity favoured by peak and trough alternations [132] and limited uptake, unless mediated by mannose 6‐phosphate receptor for lysosomal targeting, have prevented wide implementation of this strategy. Furthermore, this strategy may be difficult to implement for transcriptional factors and other regulatory molecules [133, 134].

In contrast, mRNA therapy for protein replacement bypasses most limitations of the viral gene therapies (Table 4). The advances in mRNA design and production have enabled longer half‐life and greater efficacy, achieving high levels of expression, while its transient nature provides greater control [5]. Larger transgenes can be targeted due to the large packaging capacity of LNP [47]. Developments in the field of LNP have resulted in their increased safety, which enables continued re‐dosing with acceptable safety and no noticeable immunogenicity against the transgenic protein [5, 47]. Although long‐term follow‐up studies are required to assess the benefit–risk ratio, the lack of permanent genetic alteration will be highly favourable.

**TABLE 4:** Comparing lead viral and non‐viral technologies for in vivo gene delivery.

		AAV	mRNA	Lentivirus
Technology	Large Transgene	No	Yes	Yes
	Time for efficacy	1–2 days	< 2–6 h	1–3 days
Manufacturing	Cost	High	Moderate	High
	Pre‐immunisation issues	Yes	No	No
Safety in humans	Innate immune response	Yes	Not observed in humans	Not tested in humans
	Liver toxicity	Yes	Not observed in humans	Not tested in humans
	Transient Immunosuppression	Yes	No	Not tested in humans
	Integration	Yes (low rate)	No	Yes
	Re‐injection required	No	Yes	No

The transient nature of mRNA is a double‐edged sword, providing flexibility and safety but requiring repeated dosing. Long‐term safety implications from repeated mRNA‐LNP administrations remain unknown in the absence of clinical data. Notwithstanding, lifelong dependency on this therapy presents inherent complexity. Economically, viral gene therapies incur large upfront costs, with annual spending on gene therapies predicted to reach $20.4 billion annually [135]. However, these costs are expected to be a one‐off payment for a lifelong therapy. mRNA‐LNP therapy will require sustained expenses over the patient's lifetime [136]. Manufacturing and delivery costs will need to be considered, and there is no guarantee that mRNA therapies could be marketed at a cheaper cost than expensive enzyme replacement therapies [137], especially in targeting rare diseases [138]. Repeated intravenous administrations might become a significant constraint over time.

Altogether, mRNA‐LNP could be used through various therapeutic modes:

– A long‐term cure with lifelong repeated administrations would be appropriate for many liver IMDs, avoiding the need for liver transplantation or viral gene therapy. This might be an option favoured particularly for patients with anti‐AAV neutralising antibodies.
– Using mRNA‐LNP as a rescue therapy during acute metabolic decompensation of certain IMDs (e.g., UCDs, MSUD, organic acidemias, porphyrias) is attractive due to the early peak of plasma mRNA observed in humans and the rapid expression of the transgenic protein in preclinical models. Both parameters are seen as early as 2 h post‐dosing; probably explained by the bypass of the nuclear gene expression machinery [21, 72]. The transient effect will enable return to baseline therapy following recovery. This requires rapid access to therapy and might be a logistical challenge.
– mRNA therapy could be used as a bridge to stabilise and prepare the patient for a one‐off curative approach, for example, reaching a weight threshold for a safer liver transplantation or preventing a damaging chronic liver disease as a complication of the underlying genetic IMD for AAV gene replacement or integration/editing. Depending on the context, this transient period could last from a few weeks to many years.
– mRNA‐LNP could be used as a transient therapy to control symptomatic phases of liver IMDs, which have a natural history with age‐related clinical presentation. This therapy could then be stopped when the disease is metabolically well‐controlled and re‐initiated again according to the patient's needs. An example could be citrin deficiency, which presents with an initial symptomatic neonatal presentation with neonatal intrahepatic cholestasis caused by citrin deficiency (NICCD), which could require transient mRNA therapy especially for severe cases. NICCD is followed by a post‐NICCD period when most patients are asymptomatic with strong diet specificities such as preference for protein‐ and/or lipid‐enriched food and aversion for carbohydrates [139]. These patients are unlikely to require mRNA therapy unless they present with failure to thrive and dyslipidemia in citrin deficiency (FTTDCD) or develop a damaging chronic liver disease with liver fibrosis/cirrhosis [139]. The incidence of adolescent and adult citrin deficiency (AACD) with hyperammonemic coma and neuropsychiatric complications is <10% [140]. Liver cirrhosis or pancreatitis might be observed too. These AACD complications would require mRNA‐LNP therapy.
– mRNA therapy could be used as a time‐limited adjuvant to existing therapy. This could be to correct poor metabolic control at a critical age for neurodevelopment, like in infants with phenylketonuria and prolonged inadequate control of phenylalanine levels, or pregnant women with phenylketonuria to prevent foetal complications. Equally, this could be considered when a patient is at a transient higher risk of severe acute decompensation, like in the peripartum for pregnant women affected by UCDs.

Whether mRNA therapy will become preferentially used as a long‐term cure or bridging approach is difficult to predict, especially as, in the diverse landscape of liver IMDs, each disease has specificities, and each patient may have a different perception. Options will need to be explored on a case‐by‐case basis, further guided by future clinical safety and efficacy outcomes.

Considering the prolonged journey of drug development for rare diseases, the increasing number of proof‐of‐concept studies contrasts with the limited number of clinical trials. This bottleneck needs to be addressed so that as many patients as possible could benefit from these therapies. However, the resources in setting up these complex first‐in‐human clinical trials with worldwide recruitment are limited, requiring a stagger of these clinical programmes.

Additionally, the advantages of mRNA‐LNP are being rapidly developed to mediate genome editing whereby the mRNA is encoding the editing enzyme [53]. This could result in lifelong cure with gene alteration or insertion, potentially at a specific “safe harbour” genetic locus, while the transient expression of the editing enzyme increases safety by reducing off‐target toxicity. This rapidly emerging field would provide an appealing perspective to provide lifelong cure using mRNA‐LNP. Different approaches have recently been successfully reported. A combinatorial approach with a sleeping beauty transposase delivered by mRNA‐LNP alongside an AAV‐delivered OTC transgene treated an OTC‐deficient mouse model and showed safety in non‐human primates [141]. CRISPR adenine base editor mRNA and single‐guide RNA encapsulated in LNP have cured induced pluripotent stem cell derived hepatocytes harbouring the Finnish founder ASL mutation c.1153C>T causing argininosuccinic aciduria [142]. This approach has been successfully reported in a patient with neonatal‐onset of carbamoyl‐phosphate synthetase 1 (CPS1) deficiency, a compound heterozygote mutation in the CPS1 gene. An academic team designed a personalised “*n* = 1” clinical trial with an adenosine base editor to edit one missense mutation and alleviate the clinical phenotype, allowing them to stop the protein restricted diet, halve the ammonia scavenger dose and prevent hyperammonaemia during 2 following episodes of viral infections. The base editor was delivered by 2 consecutive doses of mRNA‐LNP administered at 0.1 mg/kg/dose, with no safety‐related issue. This first‐in‐human approach was achieved within groundbreaking timelines of 7 months from the patient's diagnosis and CPS1 sequencing to first dosing [143]. Prime editing was successfully tested in a mouse model of phenylketonuria, delivering the prime editing guide RNA (pegRNA) via AAV vector and the prime editor via mRNA‐LNP. This enabled a 4% editing rate, sufficient to restore therapeutically relevant phenylalanine levels [144].

**FIGURE 4:**
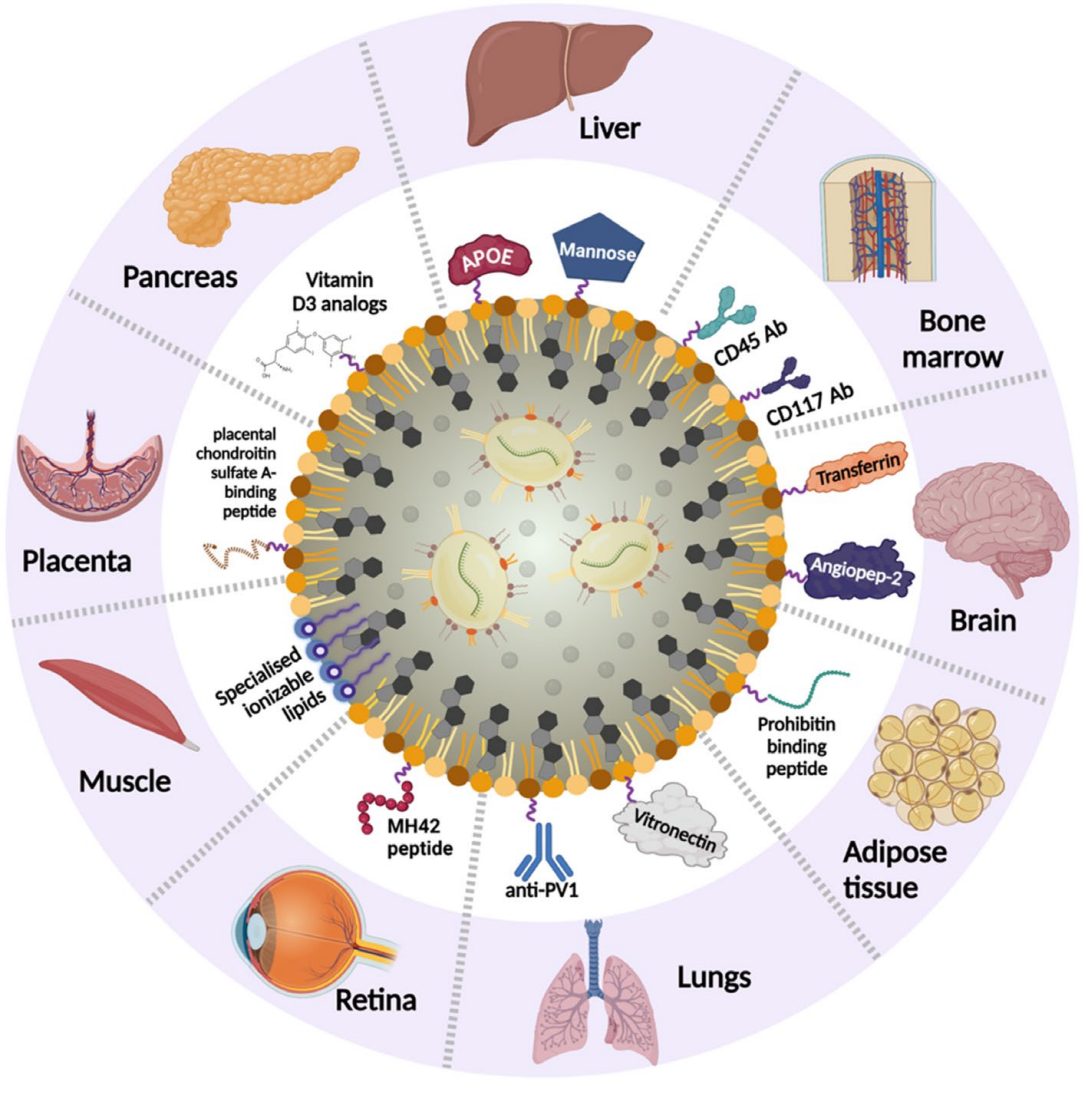
Schematic illustrating the surface functionalizations of lipid nanoparticles to enhance organ‐specific delivery. Lipid nanoparticles (LNPs) have a natural affinity for the liver due to their interactions with serum apolipoprotein E (ApoE), which facilitates hepatocyte uptake [19]. Ligands such as mannose have been used to target liver sinusoidal epithelial cells [152]. Haematopoietic stem cells (HSCs) have been reprogrammed in the bone marrow by mRNA‐LNPs decorated with the anti‐CD117 ligand [147], and in the foetal liver with anti‐CD45‐coated mRNA‐LNPs [153]. Brain‐targeted LNPs primarily employ strategies to overcome the blood–brain barrier (BBB), such as functionalization with low‐density lipoprotein receptor‐related protein 1 (LRP1)‐binding peptide angiopep‐2 [154] or anti‐transferrin receptor ligands [155], which engage receptor‐mediated transcytosis in brain endothelial cells to significantly improve brain uptake. Novel organ‐targeting peptides have been identified by phage‐display peptide screening, including a prohibitin‐binding peptide that drives selective adipocyte uptake and the MH42 peptide that directs LNPs to the retina [67, 156]. Further, chemically synthesised peptides such as the placental chondroitin sulphate A‐binding peptide were shown to enhance placental drug delivery [157]. The addition of vitamin D3 analogs to liver‐targeting LNPs markedly improves pancreatic targeting [161]. In the lungs, vitronectin‐ and anti‐PV1‐coated LNPs show selective targeting [158, 159], while muscle‐specific delivery has been achieved using specialised ionizable lipids such as D‐Lin and TCL053 [148, 160].

Overcoming the natural hepatic tropism of LNP is gradually becoming a key challenge alongside the needs to target extrahepatic organs. Many proof‐of‐concept studies have shown the use of targeted mRNA‐LNP technology for delivery to other organs such as bone marrow, skeletal muscle, heart, lung, adipose tissue, pancreas, placenta, brain and retina [19, 67, 145, 146, 147, 148, 149, 150, 151, 152, 153, 154, 155, 156, 157, 158, 159, 160, 161] (Figure 4). Specific targeting includes the incorporation of specific ligands (active targeting) or modifying lipid compositions (passive targeting) to enhance specificity to desired tissues and cells, delivered systemically and/or combined with site‐specific injections [48, 162] Active targeting includes the addition of specific ligands, peptides, or antibodies to the LNP surface while passive targeting relies on LNP physicochemical properties such as size, charge and lipid composition. Novel technologies such as nanoprimers have been developed to block liver uptake and avoid natural liver accumulation [163]. This approach uses liposomes called Nanoprimer as a decoy to limit the uptake of mRNA‐LNP of interest administered secondarily. Such potential of targeted LNP will expand the toolbox of mRNA‐LNP to applications well beyond liver IMDs.

## 7 Conclusion

mRNA‐LNP is an appealing technology for rare diseases. Its prominent efficacy in preclinical models and early safety data in clinical trials provide hope that therapeutic mRNA will become a significant step‐change for patients with IMDs. Questions on the safety and viability of therapeutic mRNA as a lifelong therapy will gradually be answered by ongoing clinical trials. Improving understanding and prevention of infusion‐related reactions is another avenue of research. However, limited resources and the necessity of global trials to target cohorts of patients with IMDs remain the bottleneck to translating numerous proof‐of‐concept preclinical studies. How this impediment will be addressed in the near future is unclear. As time elapses and experience grows, the versatility of therapeutic mRNA should become a pivotal asset for clinicians, with multiple combinations of clinical use as bridge, long‐term cure, rescue, or adjuvant therapy. The rapid development of mRNA‐LNP for gene editing/insertion should provide an alternative and potentially safer approach for a one‐off cure. Ultimately, mRNA‐LNP therapy is poised to generate a significant therapeutic impact within the landscape of liver IMDs.

## Disclosure

All authors have seen and approved the manuscript. This manuscript has not been accepted or published elsewhere.

## Ethics Statement

The authors have nothing to report.

## Conflicts of Interest

S.G. and J.B. are in receipt of research funding from Moderna Inc.
